# The 656C and 725C are two important sites in gene *STGC3* for its negative regulation on cell growth

**DOI:** 10.1080/13102818.2014.916499

**Published:** 2014-07-04

**Authors:** Suyun Li, Lili Wang, Yuanjie Xie, Xiusheng He, Zhiwei Zhang

**Affiliations:** ^a^ Cancer Research Institute, University of South China, Hengyang, Hunan, P.R. China; ^b^ Department of Pathology, Daye People's Hospital, Daye, Hunan, P.R. China

**Keywords:** nasopharyngeal, STGC3, site-directed mutagenesis

## Abstract

The aim of this study was to analyze the functional sites of the nasopharyngeal candidate tumour suppressor gene *STGC3*. Recombinant plasmid pcDNA3.1^TM^/myc-His B-*STGC3* was constructed. Site-directed mutagenesis of pcDNA3.1^TM^/myc-His B-*STGC3* plasmid at sites of C656G, C725T and T913G was induced by the Stratagene mutagenesis method. Recombinant plasmids with point mutations at C656G, C725T and T913G of gene *STGC3* were named as *STGC3*-C656G, *STGC3*-C725T and *STGC3*-T913G, respectively. CNE2 cell lines stably expressing wild and mutant *STGC3* genes were established. STGC3 expression was detected by Western Blotting and immunocytochemistry. Cell proliferation was analyzed by 3-(4, 5-Dimethylthiazol-2-yl)-2,5-diphenyltetrazolium bromide (MTT) assay and trypan blue staining. Flow cytometry analysis was used to assess apoptosis of CNE2 cells. Bax protein expression was detected by Western Blotting.

Proteins of wild-type and mutant *STGC3* genes were expressed in the cytoplasm and nucleus of CNE2 cells. Compared with the control groups, in cells stably expressing wild-type *STGC3* and *STGC3*-T913G genes, cell proliferation was significantly inhibited, whereas levels of apoptosis and Bax protein expression were significantly increased. However, the cell proliferation, apoptosis and Bax protein expression in cells stably expressing *STGC3*-C656G and *STGC3*-C725T genes were not significantly different from those in the control groups. Our results suggest that mutations at 656C and 725C, but not 913T, abolished the effects of the wild-type *STGC3* gene on CNE2 cells and that the 656C and 725C were important sites in gene *STGC3* for its negative regulation on cell growth.

## Introduction

Nasopharyngeal carcinoma (NPC) is a common malignant tumour prevalent in south-east Asia and southern China with distinctive geographical and ethnic distribution. Its incidence is associated with multiple factors, such as epstein-barr (EB) virus infection, diet, geographical and genetic factors, and involves multiple genes, multiple pathways, and multiple stages. Its etiology showed that the occurrence of NPC was related to uncontrolled cell proliferation, differentiation and disturbed apoptosis and involved the activation of proto-oncogenes and/or inactivation of tumour suppressor genes.[1–7]

The *STGC3* gene is a recently cloned nasopharyngeal candidate tumour suppressor gene (GenBank number: AY078383). The cDNA is 1271 bp located on 3p21 chromosome. The open reading frame is 438 bp, encoding a protein of 146 amino acids which is rich in leucine and serine. The molecular weight of the protein is 16 kDa. The isoelectric point is 9.96. The STGC3 protein contains a glycosylation site, a protein kinase C (PKC) phosphorylation site, a casein kinase II phosphorylation site, three myristoylation sites and a laminin G domain.[8] It plays important roles in NPC cells.[9,10] The expression of STGC3 protein was down-regulated in NPC tissues and cell lines. After transfecting the *STGC3* gene into NPC cell line, cell growth was inhibited. And the colony forming ability in soft agar was also decreased. Further, tumorigenesis in nude mice was decreased. These findings indicate that STGC3 could inhibit the growth and proliferation of NPC cells. Moreover, STGC3 overexpression increased the apoptosis rate, percentages of cells in G0/G1 phase and Bax protein expression,[9,10] whereas STGC3 overexpression decreased percentages of cells in S phase, Bcl-2 protein expression and the Bcl-2/Bax ratio. Thus the underlying mechanisms of STGC3 inhibition on NPC cell proliferation might act through promoting apoptosis and arresting cell cycle progression. Nevertheless, the functional sites in the *STGC3* gene are less studied.

The aim of this study was to screen and identify the key functional sites of gene *STGC3* by introducing point mutations in three sites: C656G, C725T and T913G; and to investigate the effects of these three mutant *STGC3* genes on the cell growth and apoptosis of CNE2 cells

## Materials and methods

### Cell lines

Poorly differentiated human nasopharyngeal squamous carcinoma cell line CNE2 was established by China Research Institute of Preventive Medicine and provided by Department of Oncology, University of South China. The cells were cultured in Roswell Park Memorial Institute (RPMI) 1640 medium (containing 10% fetal bovine serum) at 37 °C in 5% CO_2_ humidified cell incubator. 

### Reagents

The pcDNA3.1^TM^/myc-His B vector, carrying labels of myc and 6 × His, was purchased from Invitrogen. The DH5α bacteria were stored in our laboratory. Taq DNA polymerase and DNA Marker were purchased from New England Biolabs (NEB). Polymerase chain reaction (PCR) purification kit, gel extraction kit and Plasmid Miniprep kit were purchased from Omega. Lipofectamine 2000 liposome was purchased from Invitrogen. Total RNA extraction kit was purchased from Shanghai Jierui Co. Ltd. avian myeloblastosis virus (AMV) two-step PCR reaction kit was purchased from Promega. Western gel configuration kit was purchased from Biyuntian Co. Ltd. The immunohistochemistry kit was purchased from Maixin Co. Ltd. Mouse anti-human polyclonal anti-His antibody was purchased from Boster. Mouse anti-human monoclonal anti-His antibody was purchased from Invitrogen Co. Ltd. G418 was purchased from Merck. Mouse anti-human β-actin monoclonal antibody was purchased from Neomarker. Anti-Bax mouse monoclonal antibody was purchased from Cell Signaling Technology. HRP-labeled goat anti-rabbit and mouse IgG were purchased from Wuhan Boster Co. Ltd.

### Construction of recombinant plasmids with wild-type and mutant *STGC3* genes

The pcDNA3.1TM/myc-His B-STGC3 recombinant plasmid was constructed. Site-directed mutagenesis of pcDNA3.1TM/myc-His B-STGC3 plasmid at sites of C656G, C725T and T913G was induced by the Stratagene mutagenesis method. Correct plasmid was determined by sequencing (Shanghai Sangon Biological Engineering Technology & Services Co. Ltd.). Recombinant plasmids with point mutations at C656G, C725T and T913G of STGC3 gene were named as STGC3-C656G, STGC3- C725T and STGC3- T913G, respectively.

### Establishment of CNE2 cell lines stably expressing wild and mutant *STGC3* genes

The recombinant plasmids were transfected into CNE2 cell lines by Lipofectamine 2000 liposome. CNE2 cell lines stably expressing wild and mutant STGC3 genes were screened by G418.

CNE2 cells were divided into five groups, including the negative control group (cells without transfection), the vector group (cells transfected with empty vector pcDNA3.1/myc-His B), the His-STGC3 group (cells transfected with pcDNA3.1/myc-His B-*STGC3*), the His-STGC3-C656G group (cells transfected with pcDNA3.1/myc-His B-*STGC3*-C656G), the His-STGC3-C725T group (cells transfected with pcDNA3.1/myc-His B-*STGC3*-C725T) and the His-STGC3-T913G group (cells transfected with pcDNA3.1/myc-His B-*STGC3*-T913G).

### Immunocytochemistry

Expression of wild-type and mutant STGC3 in CNE2 cells was detected by an S-P immunocytochemical kit, according to the manufacturer's instructions. Briefly, cells were fixed in formaldehyde and embedded in paraffin. Sections were dewaxed, rehydrated in graded alcohols and processed before incubation with antibodies. Positive control was set up. In the negative control, the primary antibody was replaced with phosphate buffer solution (PBS). Cells with yellow or brown staining were STGC3 positive cells. Five fields at high magnification were randomly taken and positive cells were counted. Positive rate was the ratio of the number of positive cells to the number of total cells. At least 100 cells were counted. Cells without positive staining or with a positive rate less than 1% were defined as (−). Cells with a positive rate less than 25% were defined as (+). Cells with a positive rate between 25% and 50% were defined as (++). Cells with a positive rate over 50% were defined as (+++).

### Western blotting

Total proteins were extracted and separated by 12% sodium dodecyl sulfate polyacrylamide gel electrophoresis (SDS-PAGE). Then proteins were transferred onto nitrocellulose membrane. After blocking with non-fat milk, the membrane was incubated with monoclonal mouse anti-His antibody (1:500) at 4 °C overnight. After washing, the membrane was then incubated with monoclonal rabbit anti-mouse HRP conjugated antibody (1:2000) at room temperature for 1 h. Finally, the membrane was developed by enhanced chemiluminescence plus reagent. β-actin was used as an internal control.

### MTT assay

Cells were seeded into 96-well plates at a concentration of 5000 cells per well and with a volume of 200 μL per well. Then cells were cultured in a humidified incubator (37 °C, 5% CO_2_). After culturing for 3 days, 200 μL of MTT reagent (3-(4,5-dimethylthiazol-2-yl)-2,5-diphenyltetrazolium bromide, 5 mg/mL) were added and incubated at 37 °C for 4 h until purple precipitate was visible. Then the culture supernatant was removed and 150 μL dimethyl sulfoxide (DMSO) were added. The 96-well plate was oscillated for 10 min until purple precipitate was dissolved. The absorbance was measured at 490 nm on a microplate reader. Cell growth curve was generated based on these absorbance values.

### Trypan blue staining

Cells were plated into 24-well plates at a concentration of 1 × 10^4^ cells/well and cultured for eight days. At indicated time points (day 1 to day 8), cells were stained with Typan blue and cell numbers were counted. Cell growth curve was generated based on cell numbers.

### Statistical analysis

SPSS 10.0 software was used for data analysis. Comparisons between groups were tested by one-way analysis of variance (*ANOVA*). Sigmaplot 10.0 was used for mapping. A *P* value of less than 0.05 was considered statistically significant.

## Results and discussion

### Wild-type and mutant *STGC3* genes are successfully expressed in CNE2 cells

**Figure 1.**
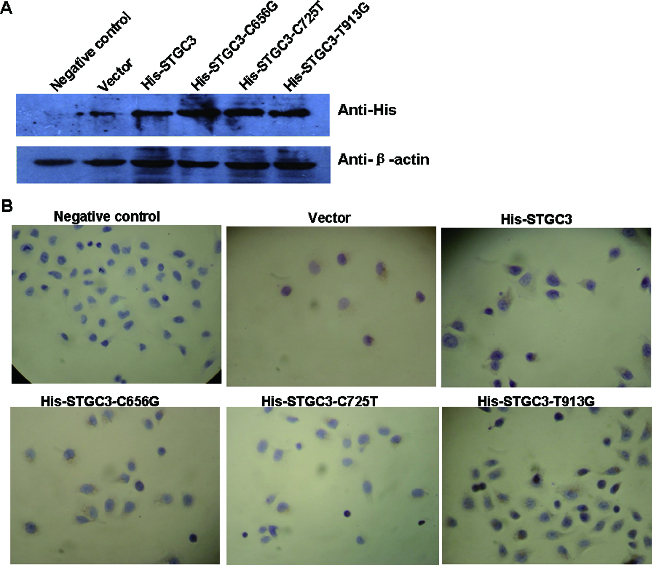
Expression analysis of wild-type and mutant *STGC3* genes in CNE2 cells. Western blotting results (A) and immunocytochemistry staining results (B) of the six groups. Cells with yellow or brown staining showed positive staining.

To determine the expression of wild-type and mutant *STGC3* genes in CNE2 cells, Western Blotting and immunocytochemistry assay were performed. For Western Blotting, total protein of each group was extracted. As shown in Figure 1(A), His-tag proteins, including His-STGC3, His-STGC3-C656G, His-STGC3-C725T and His-STGC3-T913G, were successfully expressed in CNE2 cells. This result indicates that both mutant and wild-type *STGC3* genes were expressed in CNE2 cells.

Consistently, immunocytochemistry staining showed that the His-tag proteins were located in the nucleus and cytoplasm. And His-tag protein expressions were positive in transfection groups and negative in negative control group. These data confirmed the Western Blotting result and further suggest that the transfected mutant and wild-type *STGC3* genes can be stably expressed in CNE2 cells.

### Cell growth is inhibited by His-STGC3 and His-STGC3-T913G expression while not affected by His-STGC3-C656G or His-STGC3-C725T expression

**Figure 2.**
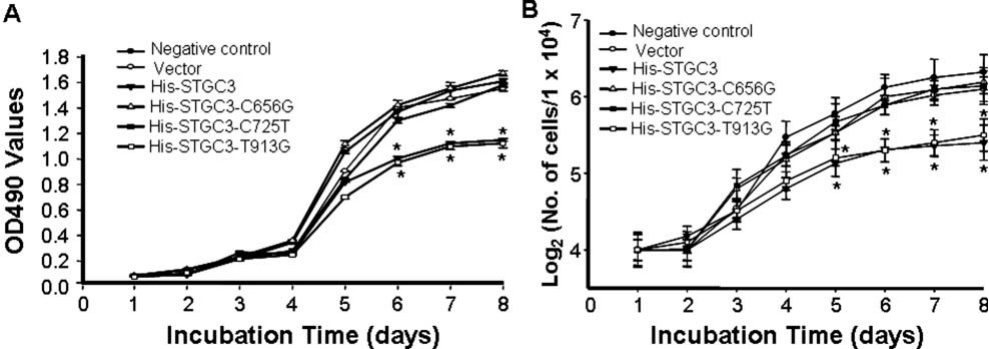
Cell growth analysis of CNE2 cells with wild-type and mutant *STGC3* gene expression. Growth curve generated by MTT assay (A). Growth curve generated by Trypan blue staining (B). Each experiment was performed three times and data were expressed as *x* ± SD. *P < 0.05.

To investigate the effect of wild-type and mutant *STGC3* gene on cell proliferation, cell growth was detected by two independent methods. First, MTT assay was performed after incubation for eight days and the result was shown in Figure 2(A). From day 1 to day 4, the OD values among the six groups were not significantly different. From day 5 to day 8, the optical density (OD) values of the His-STGC3 group and the His-STGC3-T913G group were lower than those of the negative control group, the vector group, the His-STGC3-C656G group and the His- STGC3-C725T group. Statistically, compared with the His-STGC3 group and the His-STGC3-T913G group, the OD values of the His-STGC3-C656G group and the His- STGC3-C725T group were significantly increased (*P* < 0.05). Meanwhile, there was no significant difference in OD values among the negative control group, the vector group, the His-STGC3-C656G group and the His- STGC3-C725T group (*P* > 0.05). To further verify this, cell number was counted after Trypan blue staining and a cell growth curve was generated (Figure 2(B)). The growth curve was consistent with the MTT results. Cell numbers from day 1 to day 4 were not significantly different among the six groups. However, from day 5 to day 8, cell numbers of the His-STGC3-C656G group and the His- STGC3-C725T group were significantly higher than those of the His-STGC3 group and the His-STGC3-T913G group (*P* < 0.05). Similarly, cell numbers among the negative control group, the vector group, the His-STGC3-C656G group and the His-STGC3-C725T group were not statistically different (*P* > 0.05).

Collectively, the wild-type *STGC3* gene inhibited the proliferation of CNE2 cells. Mutations at 656C and 725C, but not 913T, abolished this effect.

### Apoptosis is increased by His-STGC3 and His-STGC3-T913G expression while not affected by His-STGC3-C656G or His-STGC3-C725T expression

To determine the effect of wild-type and mutant *STGC3* gene on cell apoptosis, flow cytometry was performed. Representative results are shown in Table 1. The apoptosis rate in the negative control group, the vector group, the His-STGC3-C656G group and the His-STGC3-C725T group was 1.20% ± 0.13%, 1.50% ± 0.26%, 1.52% ± 0.20% and 1.42% ± 0.15%, respectively. There were no statistically significant differences among these groups (*P* > 0.05). However, the apoptosis rate in the His-STGC3 group and the His-STGC3-T913G group was 7.00% ± 0.57% and 7.64% ± 0.68%, both significantly higher than those in the negative control group, the vector group, the His-STGC3-C656G group and the His-STGC3-C725T group (*P* < 0.05). Meanwhile, there was no significant difference between the His-STGC3 group and the His-STGC3-T913G group. Thus, the wild-type *STGC3* gene promoted the apoptosis of CNE2 cells. Meanwhile, mutations at 656C and 725C, but not 913T, abolished this effect.

**Table 1.** Cell apoptosis results by flow cytometry analysis (*n* = 3, 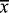 ± SD%).

Groups	Apoptosis rate (%)
Negative control group	1.20 ± 0.13
Vector group	1.50 ± 0.26
His-STGC3 group	7.00 ± 0.57*
His-STGC3-C656G group	1.52 ± 0.20
His-STGC3-C725T group	1.42 ± 0.15
His-STGC3-T913G group	7.64 ± 0.68*

*P < 0.05 vs. the negative control group.

### Bax protein expression is increased by His-STGC3 and His-STGC3-T913G expression while not affected by His-STGC3-C656G or His-STGC3-C725T expression

**Figure 3.**
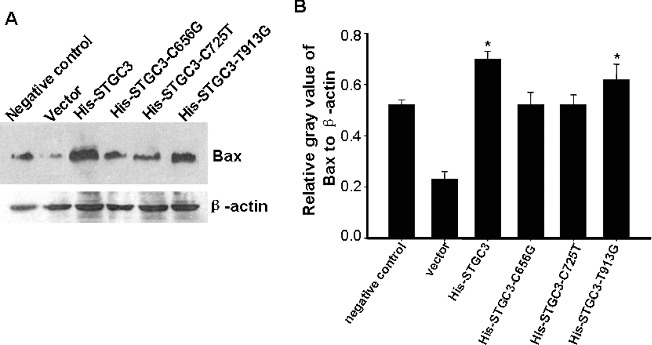
Bax protein expression analysis of CNE2 cells with wild-type and mutant *STGC3* gene expression. Representative (A) and quantitative Western Blotting results (B). The data represent *x* ± SD of three independent experiments. *P < 0.05.

To further verify the effect of wild-type and mutant *STGC3* gene on cell apoptosis, expression of Bax was detected by Western Blotting. Representative and quantitative Western Blotting results are shown in Figure 3. Bax protein expression in the His-STGC3 group and the His-STGC3-T913G group were higher than that in the other four groups (*P* < 0.05). These data suggest that the increased apoptosis rate in the His-STGC3 group and the His-STGC3-T913G group may be associated with the increase in Bax protein expression.

As a candidate tumour suppressor gene, *STGC3* gene can inhibit tumour cell growth and proliferation. And it is found that the laminin G domain may be essential for the inhibition function of STGC3.[11–17] Though there are findings about STGC3's function, the roles of specific amino acids in STGC3 are unclear.

Our study found that with point mutation of 656C into 656G at the glycosylation site, the proliferation rate of CNE2 cells was significantly higher (*P* < 0.05) than that with wild-type His-STGC3. Contrarily, the apoptosis rate and Bax expression in CNE2 cells with His-STGC3-C656G were both significantly lower than that in cells with wild-type His-STGC3 (*P* < 0.05). Glycosylation is an important form of protein modification, and is essential to maintain the biological activity of proteins. It is reported that glycosylation is related to protein stability, immunogenicity and transportation.[18] The corresponding amino acid sequence of the glycosylation motif is Asn-X-Ser/Thr, of which the residues of asparagines and serine/threonine are important in the glycosylation process. Point mutation at 656C of the *STGC3* gene abolished the glycosylation of the STGC3 protein. Furthermore, cell growth was increased and apoptosis was decreased by this mutation. These results suggest that the STGC3 protein glycosylation site is essential for the inhibitory function of the STGC3 protein.

The 725C site is an important phosphorylation site in the PKC domain of the STGC3 domain. Point mutation at 725C into 725T showed similar results as the point mutation at 656C, resulting in decrease of the cell growth inhibition and the pro-apoptotic function of the STGC3 protein. Our data suggest that the PKC phosphorylation site of *STGC3* gene is important for its biological function.

With point mutation of 913T into 913G at the casein kinase II phosphorylation site of the STGC3 protein, the cell proliferation rate, apoptosis rate and Bax expression were similar to those in cells with wild-type STGC3 protein (*P* > 0.05). However, when compared with the negative control group and the vector group, the cell proliferation rate decreased, whereas the apoptosis rate and Bax expression increased (*P* < 0.05). Protein tyrosine kinase is the catalytic protein for phosphorylation of the tyrosine residues, and is important for the cell signalling cascade, cell proliferation and differentiation process. We showed in this study that the mutated serine residue at 915 will not affect the inhibition function of STGC3 protein. This may be attributed to the fact that the phosphorylated substrate of tyrosine kinase II is specific and the change in serine base may not affect the phosphorylation. Collectively, these data indicate that the amino acid of 913T is not the key site of STGC3 protein for its inhibition function and that 656C and 725C may be the key sites for the inhibitory function of the STGC3 protein.

## Conclusions

Our study confirmed the inhibitory role of STGC3 in CNE2 cells. Furthermore, through site-directed mutation at the glycosylation site, the phosphorylation site in PKC domain and the phosphorylation site in casein kinase II domain, two sites of 656C and 725C were found important for the inhibitory function of STGC3. Our data provide experimental evidences for the investigation of the role and the underlying mechanism of STGC3 in NPC.
